# Chemoautotrophy, symbiosis and sedimented diatoms support high biomass of benthic molluscs in the Namibian shelf

**DOI:** 10.1038/s41598-022-13571-w

**Published:** 2022-06-13

**Authors:** K. Amorim, N. Loick-Wilde, B. Yuen, J. T. Osvatic, J. Wäge-Recchioni, B. Hausmann, J. M. Petersen, J. Fabian, D. Wodarg, M. L. Zettler

**Affiliations:** 1 Department of Biological Oceanography, Leibniz Institute for Baltic Sea Research Warnemünde, Rostock, Germany; 2 Centre for Microbiology and Environmental Systems Science, University of Vienna, Vienna, Austria; 3 Doctoral School in Microbiology and Environmental Science, University of Vienna, Vienna, Austria; 4 Department of Laboratory Medicine, Medical University of Vienna, Vienna, Austria; 5 Joint Microbiome Facility of the Medical University of Vienna and the University of Vienna, Vienna, Austria; 6 Department of Marine Chemistry, Leibniz Institute for Baltic Sea Research Warnemünde, Rostock, Germany

**Keywords:** Stable isotope analysis, Food webs, Molecular ecology, Ecosystem ecology, Microbial biooceanography, Element cycles

## Abstract

The molluscs *Lucinoma capensis*, *Lembulus bicuspidatus* and *Nassarius vinctus* are highly abundant in Namibian oxygen minimum zone sediments. To understand which nutritional strategies allow them to reach such impressive abundances in this extreme habitat we investigated their trophic diversity, including a chemosymbiosis in *L. capensis*, focussing on nitrogen biochemical pathways of the symbionts. We combined results of bulk nitrogen and carbon (δ^13^C and δ^15^N) and of compound-specific isotope analyses of amino acid nitrogen (AAs—δ^15^N_Phe_ and δ^15^N_Glu_), with 16S rRNA gene sequencing of *L. capensis* tissues and also with exploratory results of ammonium, nitrate and nitrite turnover. The trophic position (TP) of the bivalve *L. capensis* is placed between autotrophy and mixotrophy, consistent with its proposed symbiosis with sulfur-oxidizing *Candidatus* Thiodiazotropha sp. symbionts. The symbionts are here revealed to perform nitrate reduction and ammonium uptake, with clear indications of ammonium host-symbionts recycling, but surprisingly unable to fix nitrogen. The TP of the bivalve *L. bicuspidatus* is placed in between mixotrophy and herbivory. The TP of the gastropod *N. vinctus* reflected omnivory. Multiple lines of evidences in combination with current ecosystem knowledge point to sedimented diatoms as important components of *L. bicuspidatus* and *N. vinctus*’ diet, likely supplemented at times with chemoautotrophic bacteria. This study highlights the importance of benthic-pelagic coupling that fosters the dietary base for macrozoobenthos in the OMZ. It further unveils that, in contrast to all shallow water lucinid symbionts, deeper water lucinid symbionts rely on ammonium assimilation rather than dinitrogen fixation to obtain nitrogen for growth.

## Introduction

The Namibian shelf is one of the most productive upwelling areas in the world characterized by an enduring organic matter turnover, which leads to the formation of an oxygen minimum zone (OMZ) within the Benguela upwelling system (BUS). Shelf sediments shallower than 150 m are characterized by organic matter-rich diatom mud belts, where sulfur bacteria mats may form due to periodic formation of hydrogen sulfide (H_2_S)^1–3^. The combination of low oxygen availability and accumulations of toxic hydrogen sulfide in the sediment present extreme conditions for life^2–4^, and the central region of the OMZ supports low benthic density, biomass and highly variable dominant taxa (e.g. polychaetes, crustaceans, molluscs)^5^. However, the borders of the OMZ are home to a remarkably high biomass of mollusc species, including the bivalve *Lembulus bicuspidatus* and the gastropod *Nassarius vinctus*^6^. Furthermore, during a 2019 expedition (M157), we discovered an extraordinarily high biomass of the bivalve *Lucinoma capensis* (mean and maximum biomass of 50 g m^−2^ and 300 g m^−2^, respectively; Supplementary Table S1) in the biogenic mud neighbouring areas with the highest diffusive hydrogen sulfide fluxes at the sediment surface in the BUS^2^.

Members of the bivalve family Lucinidae have a global distribution and all species studied thus far form a nutritional symbiosis with environmentally acquired sulfur-oxidizing gammaproteobacteria, which may also play an important role in detoxifying hydrogen sulfide for their host^7–9^. Most shallow-water lucinids studied to date host symbionts from the genus *Candidatus* Thiodiazotropha^10–13^, with the exception of the mangrove lucinid *Phacoides pectinatus* which hosts a symbiont from the genus *Sedimenticola*^14^. In addition to the oxidation of reduced sulfur compounds, like hydrogen sulfide, and carbon fixation, all the various *Ca.* Thiodiazotropha symbiont species have the capacity to assimilate ammonium (NH_4_+) and to fix atmospheric nitrogen (N_2_), but vary in their ability to assimilate nitrate (NO_3_^−^) and urea, which seems to be a strategy for living in nitrogen-limited shallow-water environments (e. g. seagrass meadows and coral reefs)^10–12^. Despite growing interest in the chemosynthetic symbiosis of shallow-water lucinids, our understanding of the biology of the lucinids that inhabit the sediment of outer shelves and slopes remains very limited^15,16^. The deep water chemosynthesis-based ecosystems are often seeps and OMZs, which are environments characterized by distinct inorganic nitrogen cycling such as prominent ammonium efflux from the sediments due to reducing conditions^17^.

The molluscs *L. bicuspidatus* and *N. vinctus* also dominate the Namibian shelf sediments^6^. The bivalve *L. bicuspidatus* (Nuculanidae) is an infaunal deposit feeder with limited mobility that inhabits organic-rich sediments^6,18^. An unexpectedly high density of *L. bicuspidatus* occurs northwards of the *L. capensis* populations at a mean abundance and biomass of 515 individuals and 257 g m^−2^, respectively (Supplementary Table S1)^6^. *N. vinctus* is a highly mobile gastropod known for scavenging on dead animals and tolerance of a wide range of ambient oxygen levels^3,19^. *N. vinctus* co-occurs with *L. capensis* and *L. bicuspidatus* but lives on the sediment surface where it reaches high abundances (328 individuals m^−2^) and makes up for less of the total mollusc biomass (37 g m^−2^) (Supplementary Table S1). Therefore, despite its inhospitality, the OMZ of the BUS supports a surprisingly high biomass of benthic animals, yet little is known about their trophic ecology or the nutrient sources that allow them to achieve such high biomass under the harsh conditions in the OMZ^20^.

Stable isotopic analysis (SIA) has been widely used to study the trophic interactions of marine benthic communities^19,21–23^, and is a powerful tool for detecting nutrient sources and tracing nutrient exchanges in chemoautotrophic associations^24–26^. For example, the bulk δ^13^C values of chemosymbiotic lucinids (− 37‰ to − 23‰)^27–31^ are approximately within the bulk δ^13^C range expected when a large proportion of their carbon is derived from autotrophy with the CO_2_-fixing enzyme Rubisco I (− 35‰ to − 27‰)^24^. In contrast, non-symbiotic benthic organisms typically have δ^13^C values between − 20 and − 16‰^29,32^. Specifically, a non-symbiotic generalist deposit feeder, for instance, is expected to have δ^13^C values that resemble the signature of the local sedimentary organic matter or free-living bacteria^32^. Interestingly, the δ^13^C values of non-symbiotic benthic organisms appear to reflect signatures resulting from activities of the CO_2_-fixing enzymes PEP carboxylase and PEP carboxykinase in marine phytoplankton^33^, which may indicate that marine phytoplankton is a dietary base for non-symbiotic benthic food webs.

Bulk δ^15^N values vary from − 13 to 5‰ in chemosymbiotic organisms, and typically from 5‰ to 15‰ in non-symbiotic benthic organisms^30,32,34–39^. Bulk δ^13^C values and bulk δ^15^N values are often combined in SIA, however bulk δ^15^N values are affected by higher and more variable trophic fractionation between diet and consumers than bulk δ^13^C values (∆δ^13^_consumer-diet_ of 1.0‰). Bulk δ^15^N values are enriched by 2.3–3.4‰ in consumers compared to their diet, and more specifically by 2.5‰ ± 2.5‰ in herbivores and by 3.2‰ ± 0.4‰ in carnivores^40,41^. Therefore, one might expect the bulk δ^15^N of a deposit feeder inhabiting the Namibian shelf to reflect the δ^15^N signature of the local surface sediment nitrogen of 4.6–10.0‰^42,43^, albeit trophically enriched by 2.3–3.4‰.

However, accurate interpretation of bulk stable isotopes values requires prior characterization of the dietary base line, which is not always available^44^. Compound-specific isotope analyses of amino acid nitrogen (CSIA-AA) is a refining approach allowing the more precise assignment of trophic positions (TP) without prior base line characterization^45–47^. This is achieved by comparing the δ^15^N value of the so-called “trophic AA” glutamic acid (Glu), which is enriched by 6.4‰ ± 2.5‰ per trophic transfer, with the δ^15^N value of the so-called “source AA” phenylalanine (Phe), which reflects the isotopic composition of the dietary base line because its δ^15^N value is marginally fractionated during trophic transfer by only − 0.1‰ ± 1.6‰^45,47,48^. Because of the conservative nature of the δ^15^N value of phenylalanine in the food web, it is possible to further explore the incorporation of pelagic diatoms (sedimented over the sea floor) by members of the benthic food web by comparing the δ^15^N_Phe_ values of benthic consumers with the δ^15^N_Phe_ values of herbivorous mesozooplankton from the Namibian shelf, which has a δ^15^N_Phe_ value of 5.3‰^49^ (Supplementary Table S2).

Besides trophic interactions studies, nitrogen stable isotopes appear to reflect signatures from the dominant inorganic nitrogen source assimilated by the autotrophic base of a food web, when considering the isotopic fractionation of the specific nitrogen assimilation process. That can be done by comparing the bulk δ^15^N of an autotrophic base of a food web, with the δ^15^N values of the local dominant inorganic nitrogen sources available, which are namely dinitrogen_,_ nitrate and ammonium in both bottom and surface water off Namibia. For dinitrogen, we consider the δ^15^N to be 0.6‰ as for dinitrogen dissolved in seawater, and the maximum isotopic fractionation during dinitrogen fixation to be 2.5‰^50^. Thus, if autotrophic organisms would primarily grow on dinitrogen fixation, their bulk δ^15^N in particulate organic nitrogen (PON) would have a minimum value of − 2.1‰ (Supplementary Table S2).

The theoretical values of δ^15^N of nitrate assimilating PON in the Namibian shelf must consider two sources of extremes for δ^15^N nitrate, the upwelling water and the sediment-overlying water. The source water for upwelling in the northern BUS originates from South Atlantic Central Water (SACW), that feeds into an undercurrent off the shelf break, whose nitrate δ^15^N values vary between 5.7 and 6.7‰ near the central shelf edge (~ 170 m)^17^. From there, the upwelling water is transported towards the Namibian shelf reaching surface waters and providing comparatively isotopically lighter nitrate for pelagic phytoplankton growth, namely of diatoms. On the other hand, the suboxic or anoxic sediment-overlying water is a second isotopically heavier local nitrate source with δ^15^N values of 5.9–14.3‰, that are thought to be mainly due to isotope fractionation of nitrate during denitrification spatially overlapping with low oxygenated conditions^17,51^. The isotopic contrast between pelagic and benthic nitrate sources may be reflected in enriched δ^15^N values of PON in nitrate assimilating benthic chemoautotrophs and its consumers compared to more depleted δ^15^N at the dietary base of the pelagic food web (Supplementary Table S2). Thus, considering the isotopic fractionation to range between 4.0 and 10.0‰^50^ for nitrate assimilation by photoautotrophs, phytoplankton growing on nitrate assimilation from upwelled waters would have bulk δ^15^N for PON ranging between − 4.3 to 2.7‰ in the Namibian shelf (Supplementary Table S2). To the best of our knowledge, the fractionation factor associated with growth on nitrate in chemoautotrophic bacteria is unknown. Thus a first approximation may be to assume a similar fractionation factor as in nitrate assimilation by other prokaryotes (0.4‰ to 5.0‰)^52^. In that case, benthic chemoautotrophic bacteria growing on a local, shelf-specific nitrate source would have bulk δ^15^N for PON ranging between 0.9 and 13.9‰ at the central shelf (Supplementary Table S2).

Ammonium is abundant in the sediments of the Namibian shelf, and thus a promising inorganic nitrogen source for the benthic ecosystem. To the best of our knowledge, the δ^15^N of ammonium has not been measured for our study area. However, assuming that the δ^15^N of ammonium largely reflects the δ^15^N of particulate organic nitrogen settling to the seafloor, and that δ^15^N fractionation during ammonification of PON in organic-rich marine sediments is negligible^53–55^, we can ascribe the δ^15^N values of ammonium as the same values of sediment PON (Supplementary Table S2). Finally, knowing that the assimilation of ammonium can be associated with isotope fractionation varying from 5 to 20‰^56^, it is possible to estimate the δ^15^N of ammonium assimilating PON.

In this way, we will deduce which inorganic nitrogen source is being assimilated by the *L. capensis* symbionts by excluding the processes that cannot explain the bulk δ^15^N values in the gill tissue from *L. capensis*. Also, the more refined δ^15^N_Phe_ values will assist us on identifying dietary sources from, for instance, pelagic realms, which may strongly support benthic environment.

Our general aim was to investigate the trophic ecology of three dominant mollusc species in the Namibian shelf. We analysed bulk δ^13^C and bulk δ^15^N values as well as amino acid nitrogen specific δ^15^N_Phe_ and δ^15^N_Glu_ values of their gill (in case of *L. capensis*) and body tissues. These values are then compared to the estimated (in case of ammonium and diatoms’ PON) and reference (in case of dinitrogen, nitrate, sediment PON) δ^15^N values of the dominant organic and inorganic nitrogen pools in the OMZ including diatoms’ PON, sediment PON, dinitrogen, nitrate, and ammonium. Doing so allowed us to infer the mean trophic position (TP) of all species, the dominant inorganic nitrogen source actively used by the symbionts in case of *L. capensis*, and the main food sources of all three molluscs on the Namibian shelf^17,19,21–24,26,43^. We hypothesized that (i) the values of bulk δ^13^C, bulk δ^15^N, δ^15^N_Phe,_ δ^15^N_Glu_ and TP of *L. capensis* will be the lowest among the three species and consistent with an autotrophic lifestyle through their chemoautotrophic symbionts that grow mainly on ammonium, (ii) *L. bicuspidatus* will have intermediate values consistent with omnivory and the consumption of surface sediment PON, and (iii) *N. vinctus* would have the highest values, indicating carnivory and consistent with the scavenging of other (namely dead) animals. We combined our isotope based approach with ammonium, nitrate, nitrite and oxygen flux measurements, and metagenomic sequencing of the *L. capensis* gills to robustly unravel the nitrogen biochemical pathways and potential nitrogen sources (dinitrogen_,_ nitrate or ammonium) that sustain the *L. capensis* symbiosis in the OMZ off Namibia.

## Methods

### Sampling

**Figure 1:**
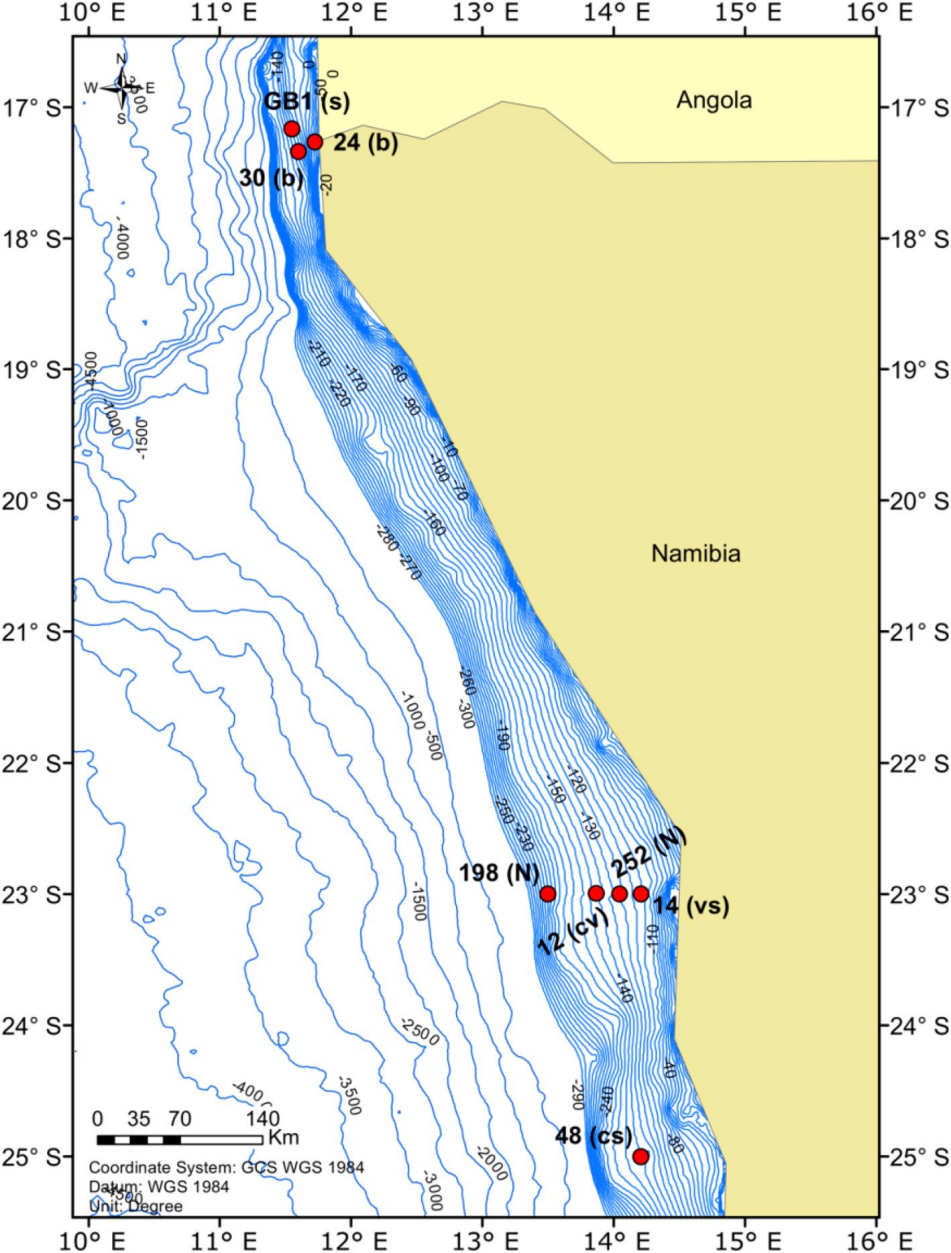
Locations of stations sampled during the M157 cruise (24, 30, 12, 14, 48). We additionally plotted the station GB1 (GeoB 1001-l-RV Meteor 6/6 1988) from Holmes et al.^43^, from which we used sediment bulk δ^15^N PON data, Stns. 252 and 198 from Nagel et al.^51^, from which we used δ^15^N of nitrate data. The letters next to the station code refer to which samples were collected or data were used in this study: s—sediment; b—*L. bicuspidatus*; c—*L. capensis*; v—*N. vinctus*; N—δ^15^N of nitrate; Figure created using ESRI ArcGIS 10.8.1. Final layout of the figure was done in Inkscape 1.1.2, www.inkscape.org.

*Lucinoma capensis, Lembulus bicuspidatus* and *Nassarius vinctus* were collected on board the RV Meteor during the EVAR Expedition M157 (18.08.2019–14.09.2019), by dredging at different stations in the Northern Benguela Upwelling System (Fig. 1, Table 1). Species were found at different stations (Stns.) possibly due to large environmental differences among stations. *L. capensis* individuals were collected in the middle and southern part of the investigation area at Stns. 12 and 48 (central and southern Namibian shelf) (Table 1). *L. bicuspidatus* were collected in the northern part at Stns. 24 and 30 (northern Namibian shelf). *N. vinctus* individuals were collected in the middle part of the investigation area at Stns. 14 and 12 (central Namibian shelf), although they can be found anywhere along the shelf. Additionally, sediment samples were collected at Stns. 14 and 48 (central and southern Namibian shelf) for bulk carbon and nitrogen stable isotope analysis. The oxygen concentration at the benthic boundary layer was measured by CTD-profiling with an attached oxygen sensor. Sediment cores were retrieved at Stns. 12, 24, 30 and 48, and ammonium and nitrate concentrations in the water overlying the sediment at these sites were measured on an auto-analyzer (QuAAtro, Seal analytical) using standard colorimetric methods by Grasshoff et al.^57^. We used sediment bulk δ^15^N PON values from Stn. GB1 (GeoB 1001-l-RV Meteor 6/6 1988) from Holmes et al.^43^. We obtained δ^15^N values of nitrate for the sediment-overlying water in the central Namibian shelf by using the data of Stns. 198 and 252 from Nagel et al.^51^, which are the extreme values measured (Supplementary Table S2).

**Table 1:** Stations sampled during the M157 cruise (24, 30, 12, 14, 48) or from published data (GB1 (GeoB 1001-l), 252, 198) which were used in this study, and the respective benthic boundary layer oxygen concentrations ([O_2_] BBL), and sediment-overlying water nitrate ([NO_3_^−^] SOW) and ammonium ([NH_4_^+^] SOW) concentrations.

Station	References	Lat (deg)	Long (deg)	Depth (m)	[O_2_] BBL (ml l^−1^)	[NO_3_^−^] SOW (μM)	[NH_4_^+^] SOW (μM)	Samples				
								*L. cap*	*L. bic*	*N. vin*	Sed	δ^15^N NO_3_^−^
GB1	Holmes et al.^43^	− 17.17	11.55	110							x*	
24	This study	− 17.27	11.72	33	2.17	29.15	1.46		x			
30	This study	− 17.34	11.60	117	2.40	37.50	1.95		x			
252	Nagel et al.^51^	− 23.00	14.21	111	0.04	9.29	4.42					x*
14	This study	− 23.00	14.05	140	0.61	–	–			x	x	
12	This study	− 23.00	13.87	150	0.73	21.80	0.86	x		x		
198	Nagel et al.^51^	− 23.00	13.50	237	1.50	16.46	0.25					x*
48	This study	− 25.00	14.20	173	2.40	24.50	4.37	x			x	

The samples refer to *L. capensis* (L. cap), *L. biscuspidatus* (L. bic), *N. vinctus* (N. vin), sediment (SED) and δ^15^N of nitrate measurements (δ^15^N NO_3_^−^) sampled during the M157 or previously published data (*).

### Bulk stable carbon and nitrogen isotope analyses

We performed bulk stable carbon and nitrogen isotope analyses with tissues of *L. capensis*, *L. bicuspidatus*, *N. vinctus* and surface sediment samples, aiming to identify the main food sources of the molluscs and also the inorganic nitrogen source assimilated by symbionts. The δ^15^N values from the sediment surface will also be used for estimating δ^15^N of ammonium, by assuming that δ^15^N fractionation during ammonification of PON in organic-rich marine sediments is negligible^53–55^. A total of three individuals of *L. capensis* from Stn. 48, 13 individuals of *L. bicuspidatus* from Stn. 24, and six individuals of *N. vinctus* from Stn. 12 were used for bulk stable isotope analysis. Specimens were collected, freshly and entirely snap frozen and stored at − 80 °C until freeze dried. *L. capensis* gills were separated from the non-symbiotic tissue. Gut content was not purged, yet we assume that the volume of the gut was negligible compared to the volume of the tissues, and gut content may not have largely affected animals’ isotopic values. The dry frozen tissues were carefully removed from the shells and ground to a homogeneous fine powder. Approximately 0.5 mg of each sample was weighted into silver weighing boats. The samples were either acidified (HCL) or non-acidified prior to bulk δ^13^C (in the following δ^13^C_Bulk_) and bulk δ^15^N (in the following δ^15^N_Bulk_) analyses (Supplementary Table S3). The non-acidified subsamples were additionally used for amino acid nitrogen specific isotope analysis.

The bulk analyses were performed at the EA-IRMS Laboratory of the Leibniz Institute for the Baltic Sea Research, Germany. Carbon and nitrogen stable isotope ratios were measured using an Elemental Analyzer (EA, Thermo Flash EA 1112), a continuous flow isotope ratio mass spectrometer (IRMS, Thermo Finnigan DeltaPlus) via an open split interface (Thermo Finnigan Conflo IV). The nitrogen (N_2_) and carbon dioxide (CO_2_) reference gases for stable δ^15^N and δ^13^C isotope analyses were calibrated against the reference materials from the International Atomic Energy Agency (IAEA): IAEA-N3 (KNO_3_) and IAEA-CH-6 (sucrose). The control of isotopic composition was done by a peptone in-house standard (Merck) after every sixth sample. The peptone standard indicated an analytical error associated with the isotope measurements of less than 0.2‰ for both isotopes.

### Amino acid nitrogen specific isotope analysis

We performed amino acid nitrogen specific isotope analysis with tissues of *L. capensis*, *L. bicuspidatus* and *N. vinctus* to better determine their trophic position and their main food source. The amino acid compound specific isotope analysis^45,46^ was performed with 10 mg subsamples of the powdered gill and non-symbiotic tissue of three *L. capensis* individuals from Stn. 48, three whole *N. vinctus* individuals from Stn. 12, three whole *L. bicuspidatus* individuals from Stn. 24. The δ^15^N values of triflouroacetyl/isopropyl ester (TFA) AA derivatives^58,59^ were measured on a Thermo GC Isolink CN with Trace 1310 (GC), coupled via a ConFlo IV combustion interface (C) to a MAT 253 (IRMS, all Thermo Fisher Scientific GmbH, Dreieich, Germany) in Warnemünde. The precision of our GC-C-IRMS measurements varied among individual AAs and sample type but the standard deviation of 2–5 analyses typically was ≤ 1.0‰. Here we show the δ^15^N values for glutamic acid (in the following δ^15^N_Glu_) and phenylalanine (in the following δ^15^N_Phe_). Details of the analysis can be found in Loick-Wilde et al.^60^.

### Biogeochemical measurements

The trophic positions (TP) were calculated according to the following equation^61^:

1 $${\text{TP}}\, = \,{1}\, + \,\left( {\delta^{{{15}}} {\text{N}}_{{{\text{Glu}}}} - \, \,\delta^{{{15}}} {\text{N}}_{{{\text{Phe}}}} \, + \,\beta } \right)/\Delta {\text{Glu}} - {\text{Phe}},$$

with β = 3.4 and ΔGlu-Phe = 7.6^62^. A TP value of 1.0 indicates autotrophy, 2.0 indicates herbivory and 3.0 indicates the first level of carnivory^46,48^. TP values within the range of 1.0–2.0 indicate mixotrophy, while values within the range of 2.0–3.0 indicate omnivory^63–66^. Depending on environmental conditions, the TP values of mixotrophic organisms may alternate between phases that are mainly “autotrophic”, with TP values of approximately 1.3, or mainly “herbivory”, with TP values of approximately 1.9^67^. Standard error (SE) in TP estimations, computed by propagation of analytical error in the individual AA determinations, did not exceed 0.2 TP.

### On board rate measurements of oxygen, nitrate, nitrite and ammonium

To support our assumptions regarding inorganic nitrogen assimilation by *L. capensis* symbionts, we have considered the results of exploratory incubations in which oxygen, nitrate, nitrite and ammonium were measured over time in the presence of each mollusc. Freshly sampled specimens were incubated on board the FS Meteor for metabolic rate measurements. Five airtight vials (20 ml) were filled with unfiltered sea-water from the benthic boundary layer at Stn. 12; seawater was collected with 10 l free-flow bottles (Hydrobios, Germany) attached to a rosette water sampler. One *N. vinctus* (13–14 mm in size) from Stn. 14 was placed in each of 3 vials; incubations were performed under approximately in situ starting conditions (10 °C). Oxygen was monitored until the first vial became anoxic (after 52 h). Another five airtight vials were filled with unfiltered water taken from cores collected at Stn. 12 by retrieving a multicore, and *L. capensis* specimens (9–12 mm in size) from Stn. 12 were incubated one individual per vial (n = 3) under approximately in situ environmental conditions (10.4 °C). Oxygen was monitored until the first vial became anoxic (after 94 h). Another four airtight vials were filled with non-filtered water removed from cores collected at Stn. 30 by retrieving a multicore. Two of the vials had one individual of *L. bicuspidatus* (size between 13 and 15.5 mm) from Stn. 30; incubations were performed under approximately in situ environmental conditions (14 °C). Oxygen was monitored until the first vial became anoxic (after 51 h). For all incubation experiments described above, one extra vial was used as blank, and another for temperature measurements. Nitrate, nitrite and ammonium concentrations were measured at the end of the incubations.

Nitrite, nitrate and ammonium concentrations were measured on an auto-analyzer (QuAAtro, Seal analytical) using standard colorimetric methods by Grasshoff et al.^57^. The nutrient samples were previously stored at 4 °C for no longer than two days. Oxygen concentrations were measured through the Optical Oxygen Meter FireStingO2 (4 channels) (PyroScience GmbH, Germany) connected with optical fibers (SPFIB) and one temperature sensor (SPFIB), using contactless oxygen sensors spots (OXSP5) attached with silicon (SPBLUE). The oxygen and the nutrients net consumption/production were calculated by subtracting the blank values from the non-blank values, divided by incubation period and multiplied by the water volume (V) using the following equation:

2 $${\text{P}} = \left( {{\text{C}}_{{{\text{Animal}}}} - {\text{C}}_{{{\text{Blank}}}} /\Delta {\text{t}}} \right) \times {\text{V}}{.}$$

The final value was divided by the Ash Free Dry Weight (AFDW) of the organism, in order to standardize the rates. We calculated wet weight by using a model of size x wet weight for each species. We further converted the wet weight values to AFDW by using the IOW-Benthic Ecology Laboratory internal conversion factors of *Nassarius incrassatus* (0.081), *Lucinoma borealis* (0.091) and *Ennucula tenuis* (0.084) for *N. vinctus*, *L. capensis* and *L. bicuspidatus*, respectively.

### Nucleic acid extraction and sequencing

Gill tissues were rapidly dissected from frozen *L. capensis* specimens. Genomic DNA (gDNA) and total RNA was extracted from approximately 300 mg tissue using the AllPrep DNA/RNA Mini Kit (Qiagen, Hilden, Germany) following the manufacturer’s procedures with the following modifications: The tissue was cut into pieces, vortexed with silanised glass beads (2 mm and 3 mm), 1 ml solution of 99.99% β-mercaptoethanol and 1% of RLT Plus buffer for 15 min. The resulting solution was then distributed into two tubes, diluted to 50% Beta merkaptor and 50% RLT Plus buffer. The extracted DNA (86.92 ng/µl ± 14.91) and RNA (182.90 ng/µl ± 89.20) were quantified with a NanoDrop ND‐1000 spectrophotometer (NanoDrop Technologies, Wilmington, Delaware, U.S.A.). cDNA was synthesized from the RNA extracts using Multiscribe reverse transcriptase, according to manufacturer’s instructions (Thermo Fisher Scientific # 4311235).

Metagenomic and amplicon sequencing were carried out by the Joint Microbiome Facility of the Medical University of Vienna and the University of Vienna under project IDs JMF-2104-13 and JMF-2104-02, respectively. The V3–V4 regions of the 16S rRNA genes were amplified from both the DNA and cDNA of RNA extracts using the 341F and 785R primers. Library preparation, sequencing, and analysis of the amplicon sequence variants (ASVs) were carried out as described by Pjevac et al.^68^. DNA libraries for metagenome sequencing were prepared using the Illumina compatible NEBNext Ultra II FS DNA Library Prep Kit. The libraries were sequenced on an Illumina NovaSeq 6000 using paired-end settings for read-lengths of 100 bp to generate a minimum of 1,000,000 reads.

Quality filtering, genome assembly, and bacterial genome binning were carried out using the scripts and workflow previously described by Osvatic et al.^13^. Only MAGs with over 90% completion (CheckM gammaproteobacterial gene set) and lower than 5% contamination were kept in subsequent downstream analysis. The taxonomy of all MAGs was assessed using GTDB version 0.3.3 (X). MAGs assigned to the genus *Ca*. Thiodiazotropha were submitted to the RAST (Rapid Annotation using Subsystem Technology; https://rast.nmpdr.org/) web server for functional annotation using the default RASTtk pipeline^69^.

### Data and analysis deposition

The data (raw reads and MAGs) have been deposited with links to BioProject accession numbers PRJNA765502 in the NCBI BioProject database (https://www.ncbi.nlm.nih.gov/bioproject/). The BioSample accession numbers for the MAGs are SAMN21561986-SAMN21561988 and SAMN21572993-SAMN21572995 are for the corresponding raw read sets.

## Results

### Bulk carbon and nitrogen isotopic signatures of *L. capensis, L. bicuspidatus, N. vinctus* and the surrounding sediments

**Figure 2:**
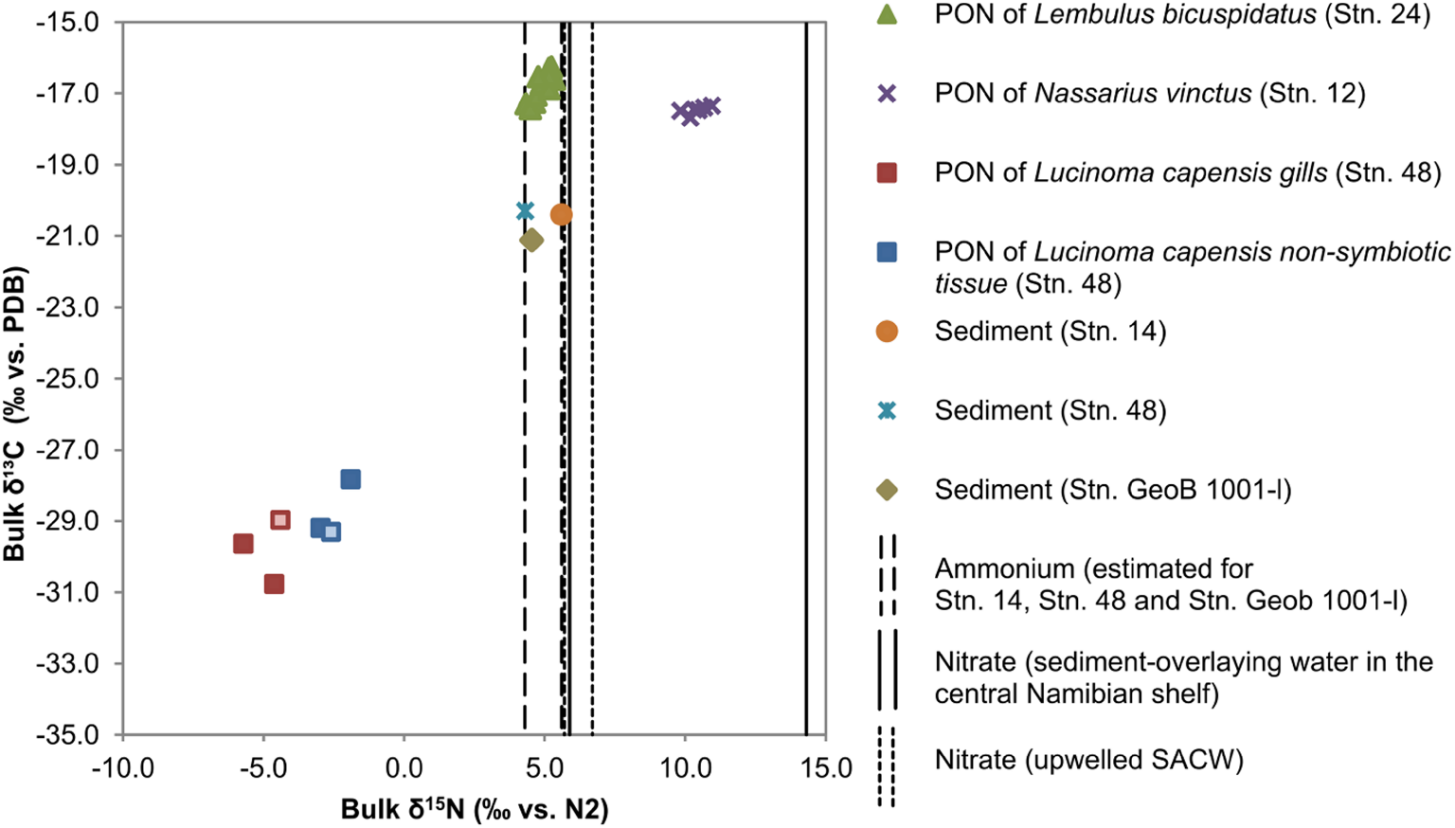
Bulk δ^15^N (‰ vs. N_2_) and bulk δ^13^C (‰ vs. PDB) signatures of *L. bicuspidatus* (n = 13), *N. vinctus* (n = 6), *L. capensis* (gills and non-gills tissue) (n = 3), and sediments from Stations 14, 48, and GeoB1001-l^43^ (n = 1). Vertical lines show the δ^15^N range of nitrate in the sediment-overlying water at the central Namibian shelf (parallel line) (Stations 198 and 252)^51^ as well as in the upwelled SACW off the Namibian shelf (dotted parallel line)^17^. In addition, vertical help lines (dashed parallel line) show the estimated δ^15^N range for ammonium calculated from PON of St. 14, Stn. 48 and Geob 1001-l (Supplementary Table S2). The light blue square (blue filled square) and the light red square (red filled square) have δ^15^N_Bulk_ values of acidified samples which were corrected by subtracting 0.3‰, the difference between acidified and non acidified samples measured in this mollusc group that were both acidified and non acidified (Supplementary Table S3).

The δ^13^C_Bulk_ values of the body and gill tissues of the three mollusc species and sediment samples spanned a wide range from − 30.8 to − 16.3‰, with the very low values for the species *L. capensis* and similar high values for the species *L. bicuspidatus and N. vinctus* (Fig. 2). Specifically, the average δ^13^C_Bulk_ value of the *L. capensis* tissues was the lowest of the three molluscs with − 29.8‰ ± 0.9‰ (n = 3) in the symbiont-housing gills and − 28.8‰ ± 0.8‰ (n = 3) in the non-symbiotic tissue (Fig. 2). In contrast, the average δ^13^C_Bulk_ values for tissues of the bivalve *L. bicuspidatus* and the gastropod *N. vinctus* were much higher at − 16.7‰ ± 0.4‰ (n = 13) and − 17.5‰ ± 0.1‰ (n = 6), respectively. The δ^13^C_Bulk_ values of the surface sediments had intermediate values but were clearly more depleted (> 1.0‰ difference) than those of the non-symbiotic mollusc species. Specifically, δ^13^C_Bulk_ values of the sediments were − 20.3‰, − 20.4‰, and − 21.1‰ at the central (Stn. 14), southernmost (Stn. 48), and northernmost stations (Stn. GeoB 1001-l)^43^, respectively.

δ^15^N_Bulk_ average values of the body and gill tissues of the three mollusc species and sediment samples also covered a wide range from − 5.7 to 10.8‰, with lowest values for the species *L. capensis*, the highest values for the species *N. vinctus*, and intermediate values for *L. bicuspidatus* and sediments (Fig. 2). Specifically, the average δ^15^N_Bulk_ values of the *L. capensis* tissues were − 4.9‰ ± 0.7‰ (n = 3) in the gills and − 2.5‰ ± 0.6‰ (n = 3) in the non-symbiotic tissues. Average δ^15^N_Bulk_ values for the bivalve *L. bicuspidatus* and the gastropod *N. vinctus* were much higher than those of *L. capensis*, at 5.0‰ ± 0.3‰ (n = 13) and 10.4‰ ± 0.4‰ (n = 6), respectively. δ^15^N_Bulk_ value of sediment at the central station (Stn. 14) was 5.6‰ and thus slightly higher than the sediment of the southernmost (Stn. 48) and northernmost stations (Stn. GeoB 1001-l), which were 4.3‰ and 4.6‰^43^, respectively. Ammonium was considered to have the same δ^15^N values as sediment PON, thus 5.6‰ at the central station, 4.3‰ at the southernmost station and 4.6‰ at the northernmost stations (Supplementary Table S2).

The reference values from literature for nitrate in the sediment-overlying water at the central Stns. 198 and 252 range from 5.9 to 14.3‰^51^, respectively. The reference values from literature for nitrate in the upwelled SACW vary in a narrow range from 5.7 to 6.7‰^17^.

In summary, the > 1‰ differences in δ^13^C_Bulk_ values between sediment and all molluscs indicate no strong dietary connection between the two, e.g. if at all, only selective parts of the sediment not reflected in the bulk δ^13^C values may be incorporated. The higher δ^15^N_Bulk_ values in *N. vinctus* compared to *L. bicuspidatus* could be due to *N. vinctus* occupying a higher trophic position or due to an isotopically heavier dissolved inorganic nitrogen source sustaining the food webs in the oxygen depleted middle part of the study area, where *N. vinctus* was sampled. This will be resolved by the δ^15^N_Phe_ and δ^15^N_Glu_ data below, although the very similar δ^13^C_Bulk_ values between both molluscs already suggest an isotopically similar dietary source for *L. bicuspidatus* and *N. vinctus*.

### Nitrogen sources and food web structure of *L. capensis, L. bicuspidatus and N. vinctus*

**Figure 3:**
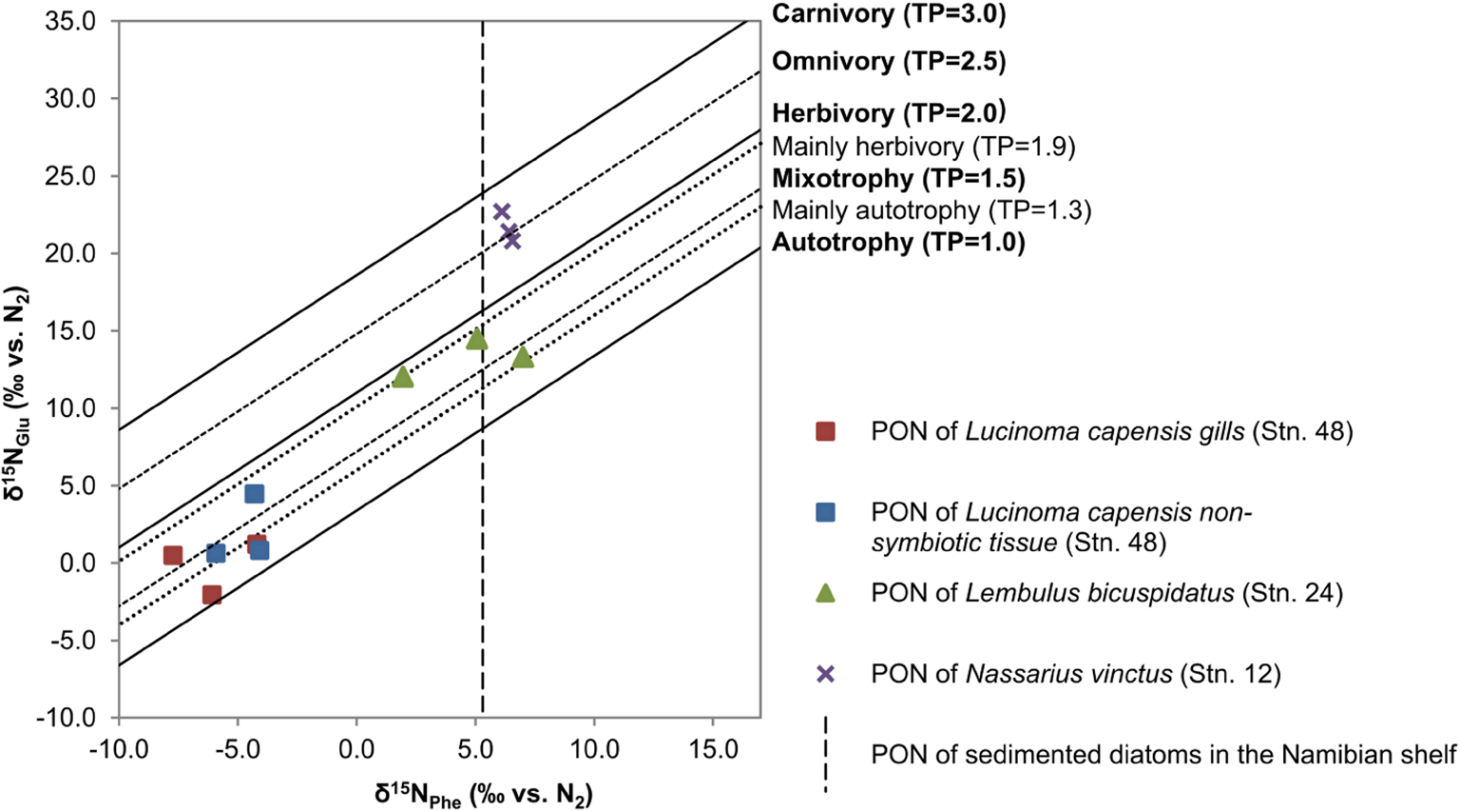
δ^15^N_Glu_ and δ^15^N_Phe_ measured for *L. capensis*, *L. bicuspidatus* and *N. vinctus*. Trophic isoclines with a slope of 1.0 and a y-intercept interval of 7.6 represent different trophic positions (TPs = 1.0, 1.5, 2.0, 2.5, and 3.0)^63–66^. The “mainly autotrophy” and “mainly herbivory” TP lines were estimated from the range of nutritional states experienced by the mixotrophic kleptoplastic gastropod *Plakobranchus ocellatus* Maeda et al.^67^. The δ^15^N_Phe_ of PON of sedimented diatoms for the Namibian shelf (dashed line) were back calculated from mesozooplankton δ^15^N_Phe_ data of Steinkopf^49^ (Supplementary Table S2).

The body tissues of all three mollusc species, and the gills of *L. capensis*, were analyzed with CSIA-AA to determine their nitrogen sources (δ^15^N_Phe_) and trophic positions in the food web (TP). The δ^15^N_Phe_ values of gill and body tissue samples revealed large differences among the species that were consistent with the δ^15^N_Bulk_ measurement patterns (Fig. 3). The δ^15^N_Phe_ values in *L. capensis* from both gill and body tissues were clearly lower than the values in *L. bicuspidatus* and *N. vinctus* and ranged from − 7.7 to − 4.0‰ with a mean value of − 6.0‰ ± 1.8‰ (n = 3) and − 4.8‰ ± 1.0‰ (n = 3) for gill and body tissues, respectively.

The δ^15^N_Phe_ values in *L. bicuspidatus* and *N. vinctus* had similar mean values of 4.7‰ ± 2.6‰ (n = 3) and 6.4‰ ± 0.2‰ (n = 3), which supports the δ^13^C_Bulk_ data indicating that both species have isotopically similar diets. Further, it shows that the differences in δ^15^N_Bulk_ values between both species (Fig. 2) cannot be explained by a change in the δ^15^N of the dietary baseline. Moreover, those values are close to the PON δ^15^N_Phe_ value estimated for sedimented diatoms consumers (5.3‰)^49^ (Supplementary Table S2) in the shelf.

The estimated TPs of the *L. capensis* gill and body tissue samples using the TP_Glu/Phe_ approach ranged from 1.1 to 1.7 (Fig. 3), thus mostly reflecting material of autotrophic and mixotrophic origin^64,65,70^. In contrast, the TP of *L. bicuspidatus* ranged from 1.4 to 1.9 (Fig. 3) reflecting mainly herbivorous and some mixotrophic feeding behaviors. Interestingly, no clear signal of predominantly carnivorous feeding behavior (TP ≥ 3.0) was found in the putative scavenger *N. vinctus*, whose TP values ranged from 2.4 to 2.7 (Fig. 3) reflecting mainly omnivorous feeding behaviors. Further, the TP comparison between *L. bicuspidatus* and *N. vinctus* shows that the differences in δ^15^N_Bulk_ values between both species (Fig. 2) can be explained by the clearly higher TP of *N. vinctus* (Fig. 3).

### The *Lucinoma capensis* gills harbour bacterial symbionts from the genus *Candidatus* Thiodiazotropha

**Figure 4:**
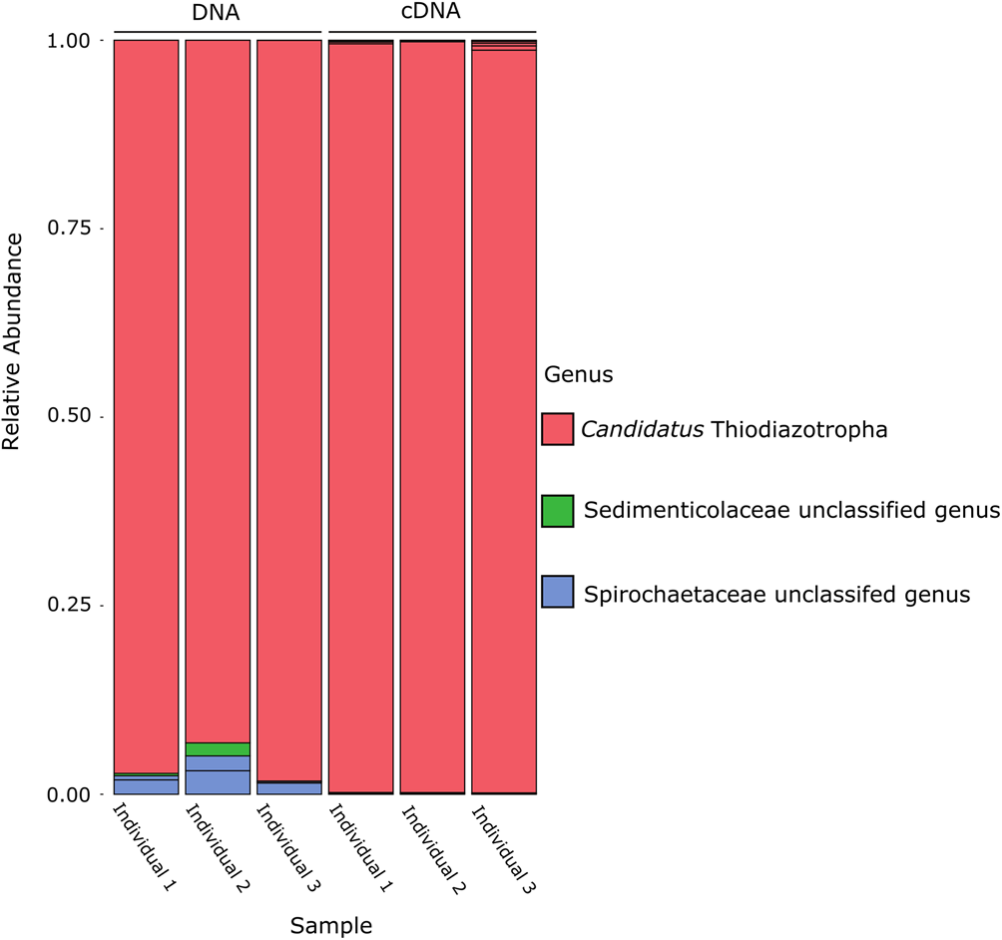
Normalized 16S rRNA sequence data of the microbiome communities in three individuals of *L. capensis*.

A single ASV from the genus *Candidatus* Thiodiazotropha dominated the *L. capensis* gill microbiome (ASV ID: ASV_6cc_309) (Fig. 4). This ASV made up between 93 and 98% of all amplicons from the DNA fraction and 98–100% of amplicons from the cDNA fractions. ASVs belonging to Spirochaetes, which have also previously been reported in lucinid gill microbiomes, were detected in the amplicon libraries from DNA, with relative abundance between 0.1 and 3%. In cDNA spirochaete ASVs made up less than 0.1% of the amplicons. Imaging methods such as FISH would be needed to demonstrate conclusively that the *Ca*. Thiodazotropha ASV detected in these animals is an intracellular symbiont like all other *Ca*. Thiodiazotropha so far investigated. However, it is likely that this highly abundant ASV, related to other lucinid symbionts, is also a gill symbiont of *L. capensis*. To further investigate the functional capabilities of this potential *Ca.* Thiodiazotropha symbiont, we sequenced, assembled, and binned the gill metagenomes of three *L. capensis* individuals. We assembled three MAGs (metagenome assembled genomes) that were assigned to the Gammaproteobacteria genus *Candidatus* Thiodiazotropha. The assembled MAGs ranged in size from 4.40 to 4.56 megabases pairs and together represented a single symbiont species based on an ANI (average nucleotide identity) threshold of 95%, and they share a 78% ANI with *Ca.* Thiodiazotropha endolucinida. All three MAGs were considered high quality with > 99% completeness and < 5% contamination.

Multiple pathways for oxidizing reduced sulfur compounds were encoded in the MAGs of the *L. capensis* symbionts (Table 2), all of which are similar to those present in previously described lucinid symbionts^10,12–14^. Energy gained from sulfur oxidation is used to fix inorganic carbon through the Calvin–Benson–Bassham (CBB) cycle, and the *L. capensis* symbiont MAGs encoded genes for two distinct types (forms I and II) of Ribulose-1,5-bisphosphate carboxylase/oxygenase. The *L. capensis* symbiont MAGs encoded genes required for heterotrophic growth, ammonium assimilation and the ability to use nitrate (narGHIJ), and nitrite (nirBD) as alternative electron acceptors like many of the previously published genomes. The genus name *Thiodiazotropha* was proposed in reference to the ability of shallow-water lucinid symbionts to fix inorganic nitrogen from the atmosphere^11,12^. However, we did not detect any genes involved in nitrogen fixation or genes involved in converting urea to ammonium; both nitrogen fixation and urea degradation are predicted functional capabilities encoded in many lucinid symbiont genomes. The absence of nitrogen fixation genes is surprising, as this pathway is present in virtually all so-far sequenced lucinid symbionts.

**Table 2:** Table with the main metabolic pathways of the MAGS *Ca.* Thiodiazotropha sp. (*L. capensis*), *Ca*. T. taylori, *Ca*. T. endolucinida, *Ca*. T. weberae, *Ca*. T. lotti.

Feature	*Ca*. Thiodiazotropha sp. (*L. capensis*)	*Ca*. T. taylori	*Ca*. T. endolucinida	*Ca*. T. weberae	*Ca*. T. lotti
**Carbon metabolism**					
CBB cycle, form I (RuBisCO)	+	+	+	+	+
CBB cycle, form II (RuBisCO)	+	−	+	−	−
Methylotrophy pathway	+	+	+	−	−
**Nitrogen metabolism**					
Diazotrophy (nif cluster)	−	+	+	+	+
Respiratory nitrate reductase (nar)	+	−	+	−	−
Periplasmic nitrate reductase (nap)	+	+	+	+	+
Copper-containing nitrite reductase (NO-forming)	+	− **	+	−	+
Nitrite reductase NADPH subunit	+	+	+	+	+
Nitric-oxide reductase	+	+	+	+	+
Nitrous-oxide reductase	+	+	+	+	+
Urea metabolism	−	+	−	+	−
Ammonium assimilation	+	+	+	+	+
**Sulfur metabolism**					
Sqr	+	+	+	+	+
Truncated SOX	+	+	+	+	+
DSR	+	+	+	+	+
DsrMKJOP complex	+	+	+	+	+
APR	+	+	+	+	+
FCC	+	+	+	+	+

Metagenomic data for all shallow-water lucinid symbionts reproduced from Osvatic et al.^13^.

+Gene(s) within the pathway were present in all the high-quality MAGs.

−Gene(s) within the pathway were absent from all the high-quality MAGs.

** These genes were only present in the high-quality MAGs of *Ca*. T. taylori associated with Stewartia floridana, from Florida, USA^13^.

### Nitrogen compound flux measurements

**Figure 5:**
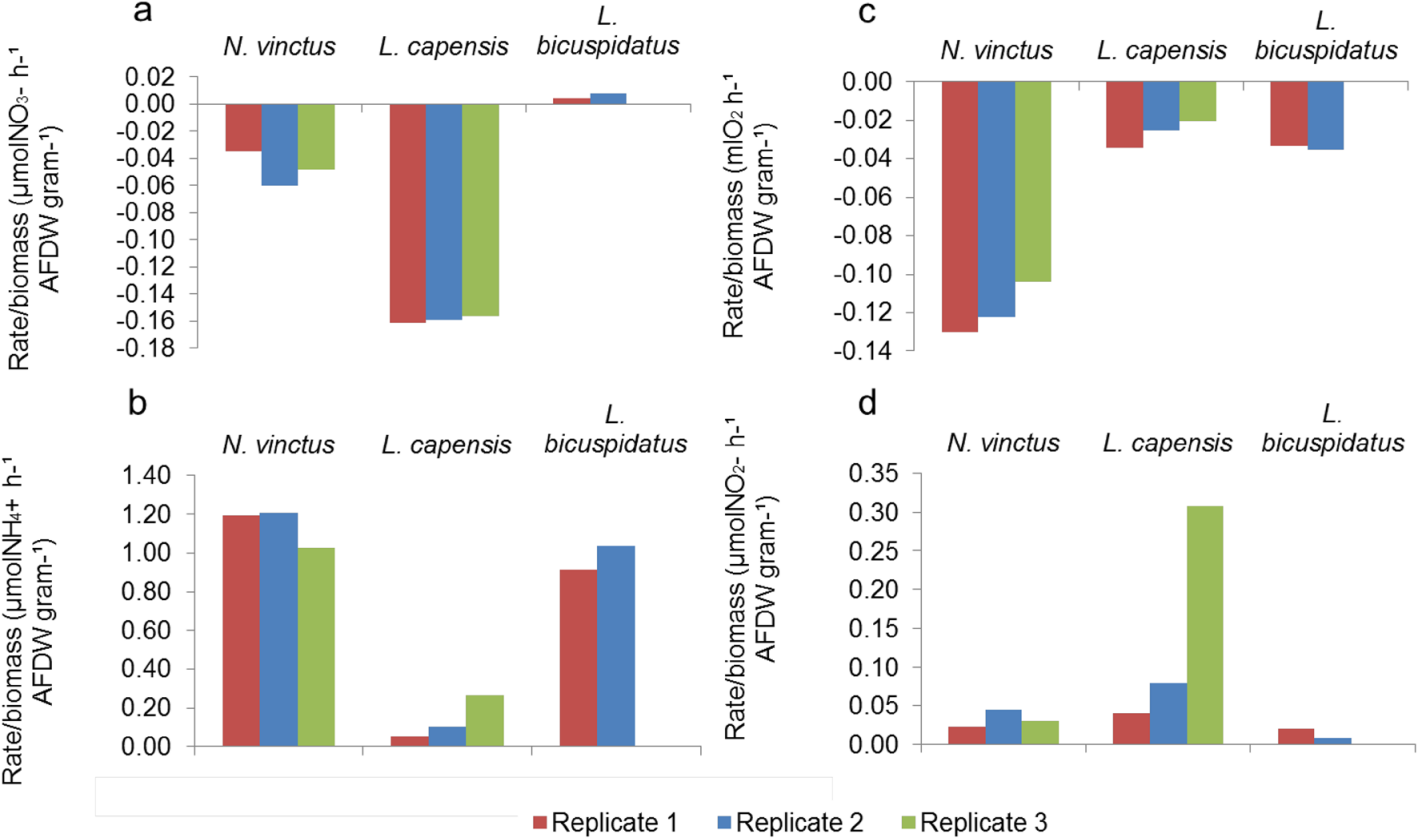
Nitrate (**a**), ammonium (**b**), oxygen (**c**) and nitrite (**d**) net consumption or net production rate (µmol X AFWDg^−1^ h^−1^ or mlO_2_ AFWD g^−1^ h^−1^) in each replicate of *N. vinctus* (n = 3), *L. capensis* (n = 3) and *L. bicuspidatus* (n = 2) incubations.

Nitrate net consumption (Fig. 5a) was highest in incubations with *L. capensis* (− 0.16 ± 0.002 µmol h^−1^ AFDW gram^−1^, n = 3) compared to *N. vinctus* (− 0.05 ± 0.01 µmol h^−1^ AFDW gram^−1^, n = 3), while *L. bicuspidatus* did not consume nitrate (0.005/0.008 µmol h^−1^ AFDW gram^−1^, n = 2). Ammonium net production (Fig. 5b), was lowest in incubations with *L. capensis* (0.14 ± 0.1 µmol h^−1^ AFDW gram^−1^, n = 3) compared to *N. vinctus* (1.14 ± 0.10 µmol h^−1^ AFDW gram^−1^, n = 3), and *L. bicuspidatus* (0.91/1.03 µmol h^−1^ AFDW gram^−1^, n = 2). Oxygen net consumption (Fig. 5c) rates were similar between *L. capensis* and *L. bicuspidatus* incubations with an average rate of − 0.03 (sd ± 0.007, n = 3) ml h^−1^ AFDW gram^−1^ for the former and values ranging from − 0.035 to 0.033 ml h^−1^ AFDW gram^−1^ for the latter, while *N. vinctus* consumed the most oxygen at an average rate of − 0.11 (sd ± 0.01, n = 3) ml h^−1^ AFDW gram^−1^. Finally, nitrite net production (Fig. 5d) was the highest in *L. capensis* incubations (0.14 ± 0.14 µmol h^−1^ AFDW gram^−1^, n = 3), compared to *N. vinctus* (0.03 ± 0.01 µmol h^−1^ AFDW gram^−1^, n = 3), and *L. bicuspidatus* (0.008/0.19 µmol h^−1^ AFDW gram^−1^, n = 2).

## Discussion

### Chemoautotrophic bacteria and/or freshly sedimented diatoms contribute to the diets of the most abundant molluscs in the BUS

The OMZ within the BUS off Namibia is characterised by periods of low oxygen availability and high levels of toxic hydrogen sulfide. Nevertheless it supports a remarkably high biomass of three benthic mollusc species: a putatively chemosymbiotic bivalve *L. capensis*, a putatively detritivorous bivalve *L. bicuspidatus*, and a putatively scavenger gastropod *N. vinctus.* Through the combination of stable isotope analyses with nitrogen compound flux measurements and metagenomic sequencing, we identified their main food sources, determine their trophic positions, and gained insights into the nitrogen metabolic pathways of *L. capensis* symbionts.

The range of δ^13^C_Bulk_ values of *L. capensis* (− 30.8‰ to − 27.8‰) fall within the range of δ^13^C_Bulk_ values (− 37‰ to − 23‰) reported for various lucinid species^27–31^, and are most comparable to the δ^13^C_Bulk_ measurements in the lucinid *Lucinoma kazani* (δ^13^C_Bulk_ of − 30.5‰ in the gills and − 28.2‰ in non-symbiotic tissues), which inhabits a cold seep environment in the Eastern Mediterranean^31^. As the difference in bulk δ^13^C between host and symbionts is normally minimal, because carbon is enriched by only 0.4–1.0‰ between each trophic level^24,32^, these depleted δ^13^C_Bulk_ values indicate lucinid biomass is made up of carbon originally derived from symbiont CO_2_-fixation through a RuBisCO enzyme^71,72^. The *L. capensis* gill symbionts belong to the genus *Ca*. Thiodiazotropha, the members of which have been described as sulfur-oxidizing chemoautotrophic symbionts of shallow-water lucinids. All three *L. capensis* symbiont MAGs encode genes for both forms I and II RuBisCO enzymes indicating the potential to convert inorganic carbon to organic carbon (Table 2). Assuming that the δ^13^C of DIC near bottom of Stn. 12 and 48 (measured during the M103 cruise) are − 0.27‰ and − 0.37‰ at 11.9 °C and 11.7 °C, respectively^73^, thus the theoretical values of δ^13^C of CO_2_ should be − 10.8‰ and − 11.0‰^74,75^ at *L. capensis’* stations. Given the bulk δ^13^C values of *L. capensis* gills (− 29.0‰ to − 30.8‰) and that the isotopic values of bulk POC of CO_2_ fixing organisms in nature are typically 10–20‰ heavier than theoretical values^71^, the discrimination against CO_2_ of the symbionts’ Rubisco would be between 28.0 and 40.0‰. Taking into account that Rubisco form I has been described to discriminate with an ε of 22–29‰ and Rubisco II with an ε of 17.8–23‰^71^, Rubisco form I is probably more strongly influencing the δ^13^C POC of the symbionts.

The ability of the *L. capensis* gill symbionts to oxidize reduced sulfur compounds and fix inorganic carbon, along with the low δ^13^C_Bulk_ and δ^15^N_Bulk_ values of the *L. capensis* tissues, suggest these bivalves rely on the chemosynthetic metabolism of their bacterial symbionts for nutrition^7–9,24,29,31^. These lines of evidence are strongly supported by the δ^15^N_Glu_ and δ^15^N_Phe_ data, which place *L. capensis* tissues at a trophic position that lies between autotrophy and mixotrophy (Fig. 3). The average TPs of *L. capensis* gills and non-symbiotic tissues (1.3 ± 0.3 and 1.4 ± 0.3, respectively) were similar to that of the kleptoplastic gastropod *Plakobranchus ocellatus* (1.3 ± 0.1) when laboratory-induced starvation of the normally algivorous animals led to an increased reliance on autotrophic nutrition from sequestered chloroplasts^67^. Mixotrophy could be an important nutritional strategy for lucinids inhabiting sediments of a highly productive Oxygen Minimum Zone, as was described in another OMZ lucinid *L. aequizonata*^76^, because feeding on particulate organic matter may allow them to survive periods of low hydrogen sulphide conditions^76^. Further studies are required to investigate the origin and importance of the particulate matter in the *L. capensis* diet.

The δ^13^C_Bulk_ and δ^15^N_Bulk_ values of the putatively non-symbiotic molluscs *L. bicuspidatus* and *N. vinctus* were much higher than that of the chemosymbiotic lucinid *L. capensis* (Fig. 2). Yet, δ^13^C_Bulk_ values alone are insufficient evidence to conclude that these two benthic molluscs are non-symbiotic, or do not consume chemoautotrophic bacteria, as the range of δ^13^C_Bulk_ values for non-symbiotic benthic organisms (− 20.0‰ to − 16.0‰)^29,32^ overlaps with the wide range of δ^13^C_Bulk_ values (− 35.0‰ to − 9.0‰) that have been reported for chemoautotrophic bacteria^24^. This is further confounded by δ^15^N_Bulk_ data, which place *N. vinctus* δ^15^N_Bulk_ values within the range of non-symbiotic benthic organisms (5‰ to 15‰), and *L. bicuspidatus* at the threshold of separating chemosymbiotic (− 13‰ to 5‰) and non-chemosymbiotic benthic organisms^30,32,34–39^. This scenario supports the statement that *N. vinctus* is not a symbiotic mollusc, however raises the question whether *L. bicuspidatus* is a facultative symbiotic mollusc. In summary, although the data consistently suggest that *N. vinctus* is a non-symbiotic mollusc, inconsistencies in the δ^13^C_Bulk_ and δ^15^N_Bulk_ values of *L. bicuspidatus* make it difficult to determine whether this species has a symbiotic or a non-symbiotic lifestyle.

Benthic molluscs inhabiting organic rich sediments, such as the seafloor of an upwelling system^2^, are often deposit feeders^77^. However, the average δ^13^C_Bulk_ values for tissues of the bivalve *L. bicuspidatus* (− 16.7‰ ± 0.4‰) and the gastropod *N. vinctus* (− 17.5‰ ± 0.1‰) were higher (> 1.0‰ difference) than those of the sediments (− 21.1‰ to − 20.3‰). Thus, there is either a decoupling between molluscs and POM, or they are feeding selectively on some components of the bulk POC in the sediments that has a different δ^13^C value—possibly corresponding to diatoms and/or sulfur-oxidizing bacteria employing Rubisco Type II^71^. However, from our knowledge and from plotting spatial data from literature, sulfur bacteria microbial mats can be patchy and are either absent or low abundant (0.2 mm^3^ cm^3^) where the molluscs species are found (Supplementary Fig. S4). Generally 67% of the production derived from the perennial upwelling in the surface waters is assumed to arrive at 100 m depth of the Namibian shelf^78^, thus constantly replenishing the diatomaceous mud with fresh diatoms. The high δ^13^C_Bulk_ values of both species match the δ^13^C_Bulk_ values of diatoms in the BUS (− 18.0‰ to − 16.0‰)^79^. Moreover, the δ^15^N_Phe_ values of *L. bicuspidatus* (4.7‰ ± 2.6‰) and *N. vinctus* (6.4‰ ± 0.2‰) are close to the δ^15^N_Phe_ of diatoms’ consumers (5.3‰), namely pelagic mesozooplankton sampled at the central Namibian shelf by Steinkopf^49^. Together this suggests that for non-symbiotic organisms, biomass of freshly sedimented diatoms seems to be a more accessible nutritional source at the diatomaceous mud belt off Namibia, although bacteria may be ingested in an opportunistic way, either by microbes annexed to the diatoms agglutinations, episymbionts living in the gills, or when there is the opportunity to feed on bacterial mats.

Our TP values of *L. bicuspidatus* (1.7 ± 0.3) place this species between herbivory (TP of 2.0) and mixotrophy (TP of 1.5), again indicating that these animals are not incorporating bulk PON from the sediment but rather fresh material that still carries the unaltered autotrophic isotopic signature. The reasoning is that the TP of degraded PON can be expected to vary from 1.9 to 2.4 due to heterotrophic microbial processes^63,80^. Thus, the TP of *L. bicuspidatus* is too low for incorporation of degraded PON with an enhanced TP, supporting that animals obtained their nutrients from selective grazing of autotrophic material with a TP of 1.

The selective incorporation of autotrophic organisms by *L. bicuspidatus* may be by collecting fresh sedimented diatoms or free living bacteria with its labial palps^77^, or by nutrition from chemoautotrophic epibionts from the gills^81^. Interestingly, the TP range of *L. bicuspidatus* (1.4–1.9) was a surprisingly close fit to the TP range of the kleptoplastic gastropod *P. ocellatus*, which was 1.9 during algivory in the wild and 1.3 when reliant on chloroplasts for nutrition during lab-induced starvation^67^. Although there is no evidence yet of a symbiotic association between autotrophic bacteria and *L. bicuspidatus*, it is interesting to speculate that an undiscovered nutritional symbiosis with chemosynthetic bacteria could partially explain the low TP values of *L. bicuspidatus* tissue.

The TP of *N. vinctus* (2.6 ± 0.2) indicates an omnivorous lifestyle. At least 50% of the *N. vinctus* diet is from a heterotrophic source and the other 50% from an autotrophic source which, based on our δ^13^C_Bulk_ and δ^15^N_Phe_ results, are likely to be diatoms. However, the δ^15^N_Phe_ (6.4‰) of *N. vinctus* is slightly higher than that of herbivorous mesozooplankton from the central Namibian shelf (5.3‰)^49^ (Supplementary Table S2), which suggests that part of their diet comprises benthic autotrophs, i.e. heavier δ^15^N nitrate assimilating organisms at the base of the food web in the central Namibian shelf. Salt marshes mud-floating snails are known to feed on diatoms conspicuous at mud surface^82,83^, with the facultative carnivore *Illyanassa obsolete*^84^ potentially feeding upon annelids^85^. Similarly, *N. vinctus* has an extended foot that allows it to float at the surface of the diatoms mud. This probably allows it to feed on recently sedimented diatoms (δ^15^N from − 4.3 to 2.7‰) mixed with heavier δ^15^N nitrate (5.9‰ to 14.3‰) assimilating benthic chemoautotrophic bacteria (assumed to have a δ^15^N from 0.9 to 13.9‰ if a similar fractionation factor range for nitrate assimilation applies in all prokaryotes) (Supplementary Table S2) floating at the mud surface, while also supplementing its diet with carnivory (TP of 2.6 ± 0.2).

### Ammonium cycling plays an important role in the dietary ecology of the deep water lucinid *Lucinoma capensis*

Sulfur-oxidising bacteria from the genus *Ca*. Thiodiazotropha are associated with lucinids from shallow-water habitats around the world and all *Ca*. Thiodiazotropha MAGs described to date encode the *nif* cluster of genes for fixing dinitrogen; a strategy critical for living in oligotrophic and nitrogen-limited ecosystems such as seagrass meadows and coral reefs^10–14^. However, none of the genes required for performing dinitrogen fixation were present in the MAGs of the *Ca*. Thiodiazotropha associated with *L. capensis*; this is unlikely to be a technical artefact as all three MAGs have a high level of completeness (> 99%). Furthermore, given that the δ^15^N of dissolved dinitrogen is 0.6‰ and the maximum isotope fractionation of dinitrogen fixation is 2.5‰^50^, the depleted δ^15^N_Bulk_ (− 4.9‰ ± 0.7‰) values in *L. capensis* gills are thus inconsistent with the symbionts obtaining nitrogen from dinitrogen fixation and point instead to alternative N-sources like nitrate or ammonium.

The δ^15^N nitrate in the sediment-overlying water in the central Namibian shelf ranges from 5.9 to 14.3‰^51^, and the isotopic fractionation of nitrate assimilation by prokaryotes from 0.4 to 5.0‰^52^. Accordingly, PON resulting from nitrate assimilation processes have δ^15^N_Bulk_ values varying from 0.9 to 13.9‰. Thus, the δ^15^N_Bulk_ (− 4.9‰ ± 0.7‰) values of the *L. capensis* gills remain lower than expected to conclude that nitrate is a significant source of nitrogen for the symbiosis. Additionally, our inability to detect genes for assimilatory nitrate reductases in the MAGs of the *L. capensis* symbionts further supports the exclusion of nitrate assimilation as the main process responsible for obtaining nitrogen for growth.

Assuming that the δ^15^N of ammonium largely reflects the δ^15^N of particulate organic nitrogen settling to the seafloor, and that δ^15^N fractionation during ammonification of PON in organic-rich marine sediments is negligible^53–55^, we can deduce the δ^15^N of the ammonium pool from sediment PON δ^15^N values, thus varying from 4.3 to 5.6‰ in the study area sediments (Supplementary Table S2). Taking into account an in situ ammonium concentration of 4 µM in the water collected from cores at Stn. 48 (Table 1) and that ammonium assimilation can have an isotope effect varying from 5 to 20‰ in marine microbes under an ambient ammonium concentration of approximately 4µM^58^, the δ^15^N_Bulk_ (− 4.9‰ ± 0.7‰) of *L. capensis* gills lead us to propose that the *L. capensis* symbionts rely on ammonium assimilation. This is clearly supported by the presence of the genes required for ammonium assimilation in the *L. capensis* symbiont MAGs (Table 2) and the threefold lower ammonium release rates in the *L. capensis* incubations compared to *L. bicuspidatus* and *N. vinctus,* a difference which is unlikely to be explained by a lower metabolic rate since oxygen consumption rates of *L. capensis* were similar to the bivalve *L. bicuspidatus*.

**Figure 6:**
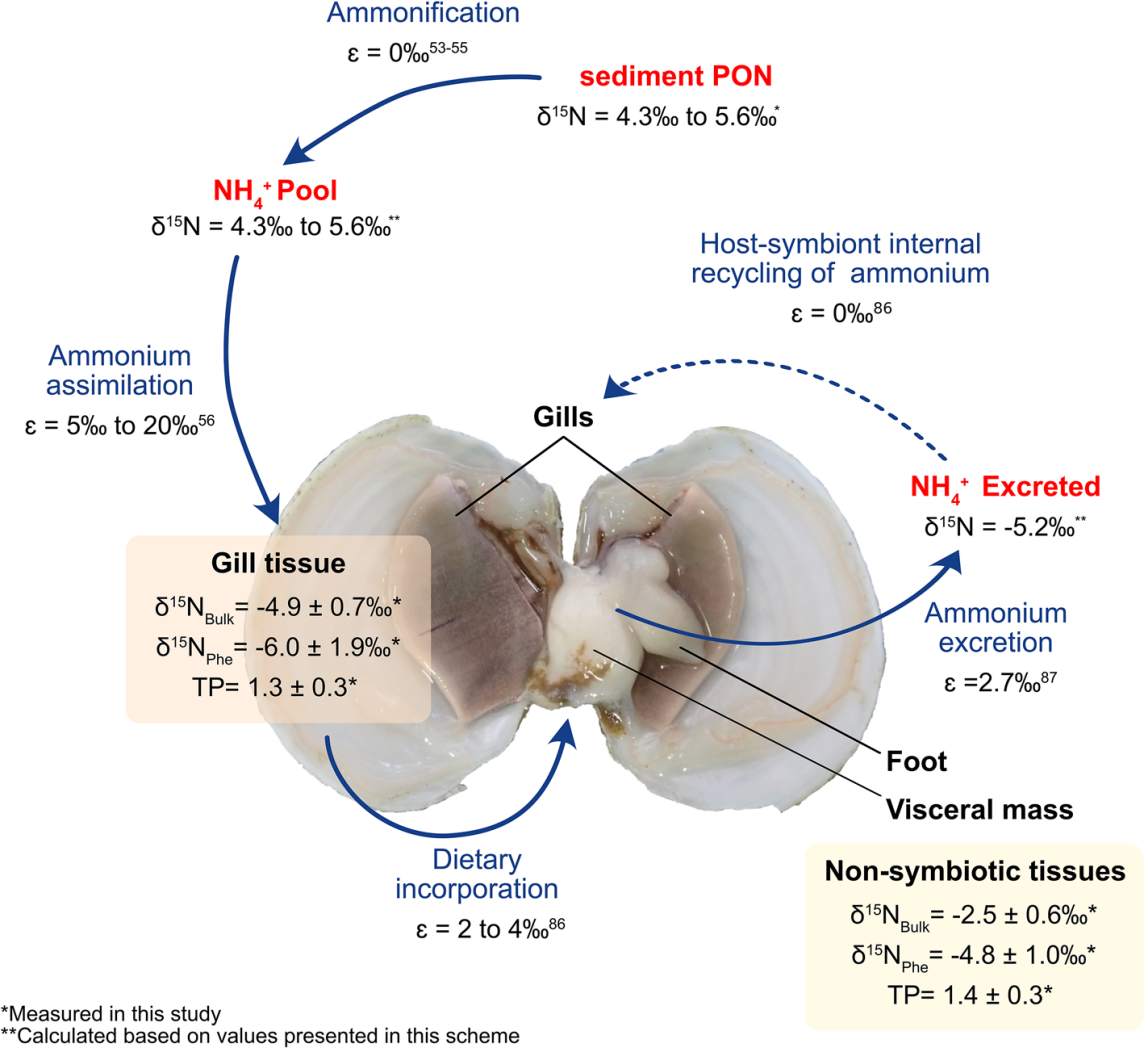
Schematic depiction of the ammonium assimilation and excretion pathways of *Lucinoma capensis* and its symbionts, based on the δ^15^N of different N pools. δ^15^N_Bulk_, δ^15^N_Phe_ and δ^15^N_Glu_ were measured in this study (*). The δ^15^N of ammonium pool and ammonium excreted were calculated in this study based only on numbers provided in this scheme (**). We assumed that δ^15^N fractionation during ammonification of PON in organic-rich marine sediments is negligible^53–55^. The fractionation factor for ammonium assimilation is from Hoch et al.^56^. The fractionation factor for diet incorporation and internal ammonium assimilation in a closed system (recycling) are from Montoya^86^. The fractionation factor for ammonium excretion is from Checkley and Miller^87^. Figure made in Inkscape 1.1.2, www.inkscape.org.

An alternative non-mutually exclusive possibility is that nitrogenous waste products excreted by *L. capensis* in the form of ammonium can be taken up and “internally recycled” by the symbionts^24^. Assuming that ammonium does not accumulate, in which case no isotope fractionation occurs^86^, “internal recycling” could easily explain the low δ^15^N_Bulk_ (− 4.9‰ ± 0.7‰) and possibly also the δ^15^N_Phe_ (− 6.0‰ ± 1.8‰) values in the symbiotic gill tissue. Considering that the non-symbiotic tissue has a δ^15^N_Bulk_ value of -2.5‰, and the fractionation factor of ammonium excretion is 2.7‰^87^, the ammonium produced by the bivalve would have a δ^15^N of − 5.2‰ (Fig. 6). This value fits well to the bivalves gills δ^15^N_Bulk_ when considering that fractionation does not take place in closed systems when the reaction goes to completion^86^.

It is important to highlight that an external source of nitrogen is still necessary for net growth^88^, and the *L*. *capensis* symbionts may be using both the environmental and host-excreted ammonium. The ammonium produced by *L*. *capensis* was low (0.14 µmol h^−1^ AFDW gram − 1 ± 0.1 (n = 3)) compared to the Mediterranean lucinid *Loripes orbiculatus*, which has a minimum average ammonium net excretion of about 5 µmol h^−1^ SFDW g^−1^^89^. *L*. *orbiculatus* inhabits nitrogen-limited seagrass meadows in the Mediterranean where there is negligible ammonium available in the environment, so host and symbiont must thus rely on the thermodynamically expensive dinitrogen fixing process to meet their nitrogen requirements^89^. The BUS, on the other hand, is a productive upwelling region with high organic matter turnover, resulting in the formation of an OMZ that is rich in ammonium^2^. *L*. *capensis* can therefore exploit the high availability of ammonium in the reducing sediments of an OMZ as a reliable source of nitrogen^39,90^. These critical differences in the habitats of *L*. *capensis* and *L*. *orbiculatus* are likely to be key factors driving the differences in nitrogen metabolism seen between shallow and deeper lucinids’ symbionts.

### The potential use of nitrate as an electron acceptor by an OMZ chemo-symbiotic mollusc

Nitrate consumed by *L*. *capensis* was at least threefold higher than that of both *N*. *vinctus* and *L*. *bicuspidatus*. Our findings further show that nitrate reduction is coupled with elevated nitrite net production, which is consistent with the activity of genes encoding the dissimilatory nitrate-reductase complex (narGHIJ) and the absence of the assimilatory pathway in the *L*. *capensis* symbiont MAGs (Table 2). These findings suggest that *L*. *capensis* symbionts use nitrate as an alternative electron acceptor^90^. Indeed, symbionts of *L. aequizonata,* which also inhabits an OMZ, are capable of nitrate respiration even under oxic conditions, and produce nitrite under aerobic conditions^91^. Nitrite net production rates in *L*. *capensis* incubations were highly variable and although we had only few replicates, they appeared to correlate with the oxygen concentration present during the incubation; the highest nitrite net production rate (0.3 µmol l^−1^ AFDW day^−1^) was observed in the vial with the highest oxygen concentrations during the whole incubation. The initial oxygen concentration in this vial was 3.23 ml l^−1^ and 2.1 ml l^−1^ at the end, while the other replicates reached anoxia at the end of their incubations. As genes encoding nitrite reductases were present in the *L*. *capensis* symbiont MAGs, this discrepancy may be explained by the inhibitory effects of high oxygen concentrations on nitrite reductase activity^92^. Future incubations with additional clam individuals or with physically enriched symbionts under defined conditions could be used to test this hypothesis.

## Supplementary Information

Supplementary Information.

## Data Availability

The metagenomic and amplicon sequencing data have been deposited with links to BioProject accession numbers PRJNA765502 in the NCBI BioProject database (https://www.ncbi.nlm.nih.gov/bioproject/). The BioSample accession numbers for the MAGs are SAMN21561986-SAMN21561988 and SAMN21572993-SAMN21572995 are for the corresponding raw read sets. All other datasets used during the current study are available from the corresponding author on reasonable request.
