# Supplementary material for: Chemoautotrophy, symbiosis and sedimented diatoms support high biomass of benthic molluscs in the Namibian shelf
Source: Sci Rep. 2022 Jun 13;12:9731. doi: 10.1038/s41598-022-13571-w (PMC9192762; doi:10.1038/s41598-022-13571-w)
Supplement: Supplementary file 1 — Supplementary Information. [file 41598_2022_13571_MOESM1_ESM.pdf]

Supplementary material

Supplementary Table S1 – Table with latitudinal range distribution, depth range distribution, mean and maximum abundance, and mean and maximum biomass of *L. capensis*, *L. bicuspidatus* and *N. vinctus* sampled at the Namibian shelf during four cruises performed in 2004 (AHAB-8), 2008 (MSM7), 2011 (MSM18/5) and 2019 (M157).

Species	Latitudinal distribution (deg)	Depth distribution (m)	Mean (Max.) abundance (ind.m^{-2})	Mean (Max.) biomass (g m^{-2})
L. capensis	-25.00 – -17.26	31 – 362	45 (215)	51 (300)
L. bicuspidatus	-18.38 – -17.25	25 – 151	515 (2532)	257 (1389)
N. vinctus	-25.00 – -17.25	25 – 280	328 (1877)	37 (228)

Supplementary material

Supplementary Table S2 - Summary of $\delta^{15}N$ values of nitrogen sources and particulate organic nitrogen (PON) in the Namibian shelf (<300 m), from literature references or deduced from the process-specific nitrogen isotope fractionation factor (ε). Values deduced are given in italic. The $\delta^{15}N$ values of nitrate used by diatoms are considered to be the $\delta^{15}N$ values of the upwelled SACW[1]. The $\delta^{15}N$ values of nitrate at the sediment-overlaying water at the central Namibian shelf are the measurements near bottom of Stns. 192, 195, 198, 202, 204, 206, 225, 230, 231, 243, 252 (n=11) from Nagel, et al.[2]. The $\delta^{15}N_{Phe}$ of mesozooplankton were calculated by averaging mesozooplankton $\delta^{15}N_{Phe}$ measured by Steinkopf[3] at the central Namibian shelf. References: Prokopenko et al.[4], Prokopenko et al.[5], Robinson et al.[6], Sigman and Fripiat[7], Nagel et al.[1], Nagel et al.[2], Granger et al.[8], Hoch et al.[9], Checkley and Miller[10], Montoya[11], McMahon and McCarthy[12], Steinkopf[3], this study*

N Source	N Process	N Product	ε	$\delta^{15}N$ of N Source	$\delta^{15}N$ of the N Product via ε
Bulk sediment PON in the Namibian shelf	Ammonification	NH_4^+ sediment-overlaying water at the Namibian shelf	0.0‰[4,5,6]	4.3 to 5.6‰*	*4.3 to 5.6‰*
N_2	N_2 Fixation	Bulk PON	2.5‰[7]	0.6‰[7]	*-2.1‰*
NO_3^- SACW upwelled	NO_3^- uptake	Bulk PON of diatoms	4.0‰ to 10.0‰[7]	5.7‰ to 6.7‰[1]	*-4.3‰ to 2.7‰*
NO_3^- sediment-overlaying water at the central Namibian shelf	NO_3^- uptake	Bulk PON benthic base source	0.4 ‰ to 5.0 ‰[8]	5.9 to 14.3‰[2]	*0.9‰ to 13.9‰*
NH_4^+ sediment-overlaying water at the Namibian shelf	NH_4^+ assimilation	Bulk PON benthic base source	5‰ to 20‰[9]	*4.3 to 5.6‰*	*-15.7‰ to 0.6‰*
Bulk PON of non-symbiotic tissue	Ammonia excretion	NH4+ excreted by the host	2.7‰[10]	-2.5‰*	*-5.2‰*

NH_4^+ excreted by the host	NH_4^+ assimilation	Bulk PON of symbionts	0‰[11]	*-5.2‰*	*-5.2‰*
Phenylalanine – PON of diatoms	Trophic transfer	Phenylalanine – PON of mesozooplankton	0‰[12]	*5.3‰*	5.3‰[3]
Phenylalanine - PON of fresh sedimented diatoms	Trophic transfer	Phenylalanine PON of benthic consumer	0‰[12]	*5.3‰*	*5.3‰*

1 Nagel, B. *et al.* N-cycling and balancing of the N-deficit generated in the oxygen minimum zone over the Namibian shelf-An isotope-based approach. *Journal of Geophysical Research: Biogeosciences* **118**, 361-371, doi:10.1002/jgrg.20040 (2013).

2 Nagel, B. *et al.* Nutrients and δ^{15}N measured in water samples in the oxygen minimum zone over the Namibian shelf during the Meteor campaign M76-2 in 2008. *PANGAEA* https://doi.org/10.1594/PANGAEA.892369 (2018).

3 Steinkopf, M. Trophische Strukturen des Mesozooplanktons im Benguela Auftriedsgebiet vor Namibia (Universität Rostock, 2018).

4 Prokopenko, M. G., Hammond, D. E., & Stott, L. Lack of isotopic fractionation of δ ^{15}N of organic matter during long-term diagenesis in marine sediments, ODP Leg 202, Sites 1234 and 1235. In *Proceedings from the Ocean Drilling Program*(eds. R. Tiedemann, A. C. Mix, C. Richter and W. F. Ruddiman), 22, (2006).

5 Prokopenko, M. G. *et al*. Nitrogen cycling in the sediments of Santa Barbara basin and Eastern Subtropical North Pacific: Nitrogen isotopes, diagenesis and possible chemosymbiosis between two lithotrophs (Thioploca and Anammox)—"riding on a glider". *Earth and Planetary Science Letters* **242**, 186-204 (2006).

6 Robinson, R. S. *et al.* A review of nitrogen isotopic alteration in marine sediments. Paleoceanography **27**, http://dx.doi.org/10.1029/2012PA002321 (2012).

7 Sigman, D. & Fripiat, F. Nitrogen isotopes in the Ocean. In *Encyclopedia of Ocean Sciences* (Third Edition) (eds J. Kirk Cochran, Henry J. Bokuniewicz, & Patricia L. Yager) 263:268 (Academic Press, 2019).

8 Granger, J., Sigman, D. M., Rohde, M. M., Maldonado, M. T., & Tortell, P. D. N and O isotope effects during nitrate assimilation by unicellular prokaryotic and eukaryotic plankton cultures. *Geochimica et Cosmochimica Acta*, **74**, 1030-1040 (2010).

9 Hoch, M. P., Fogel, M. L. & Kirchman, D. L. Isotope fractionation during ammonium uptake by marine microbial assemblages. *Geomicrobiology Journal* **12**, 113-127, doi:10.1080/01490459409377977 (1994).

10 Checkley, D. M. & Miller, C. A. Nitrogen isotope fractionation by oceanic zooplankton. *Deep Sea Research Part A. Oceanographic Research Papers* **36**, 1449-1456, doi:https://doi.org/10.1016/0198-0149(89)90050-2 (1989).

11 Montoya, J. P. Natural abundance of ^{15}N in marine planktonic ecosystems. *Stable Isotopes in Ecology and Environmental Science*, 176-201, doi:https://doi.org/10.1002/9780470691854.ch7 (2007).

12 McMahon, K. W. & McCarthy, M. D. Embracing variability in amino acid $\delta^{15}N$ fractionation: mechanisms, implications, and applications for trophic ecology. Ecosphere 7, e01511, doi:https://doi.org/10.1002/ecs2.1511 (2016).

Supplementary material

Supplementary Table S3 – Table with $\delta^{15}N_{Bulk}$ values of the Bulk Stable Isotope Analysis performed with *L. capensis, L. bicuspidatus* and *N. vinctus*. Values marked with * are $\delta^{15}N_{Bulk}$ values of acidified sample which were corrected by subtracting 0.3‰, the mean difference between acidified and non acidified samples observed in *L. capensis* tissues that were both acidified and non acidified.

Samples	Individual	$\delta^{15}N_{Bulk}$ (‰)		$\delta^{13}C_{Bulk}$ (‰)	
		non acidified	acidified	acidified	non acidified
Lucinoma capensis non-symbiotic tissue (Stn. 48)	1	-1.90*	-2.20	-27.82	
	2	-2.98	-3.29	-29.20	-28.87
	3	-2.60	-2.80	-29.30	-29.22
Lucinoma capensis gills (Stn. 48)	1	-4.40*	-4.67	-28.97	
	2	-5.71	-6.24	-29.65	-29.29
	3	-4.62	-4.83	-30.76	-30.65
Lembulus bicuspidatus (Stn. 24)	1	4.33	4.32	-17.29	-16.98
	2	4.66	4.48	-17.25	-16.86
	3	4.49	4.13	-17.43	-16.89
	4	4.70	4.37	-17.05	-16.34
	5	4.88	4.95	-16.74	-16.33
	6	5.35	5.25	-16.60	-15.80
	7	5.21	5.22	-16.31	-16.18
	8	5.16	5.06	-16.87	-16.75
	9	5.25	4.94	-16.26	-15.75
	10	5.16	5.10	-16.29	-16.38
	11	5.25	4.92	-16.41	-16.16
	12	5.17	5.11	-16.50	-16.13
	13	4.77	4.73	-16.53	-16.40
Nassarius vinctus (Stn. 12)	1	9.83	9.44	-17.49	-16.53
	2	10.68	10.22	-17.40	-16.51
	3	10.32	9.88	-17.45	-16.99
	4	10.95	10.75	-17.34	-16.75
	5	10.47	10.14	-17.47	-16.93
	6	10.19	9.90	-17.68	-17.38

Supplementary material

Supplementary Figure S4 – Map with reports of visual occurrence or absence of large sulfur bacteria from Neumann et al.[1], Brüchert et al.[2] and M157 cruise (present study), and stations where molluscs were sampled. When biovolume or biomass data were available, we reported low abundance of large sulfur bacteria for values equal or lower than 0.4 $mm^3.cm^{-3}$ (0.2 – 0.4 $mm^3.cm^{-3}$) (data from M157 cruise) or 6.8 $g.m^{-2}$ (0.3 – 6.8 $g.m^{-2}$) (data from Brüchert et al.[2]). The higher values were considered intermediate or high abundance (0.8 – 2.1 $mm^3.cm^{-3}$; 13.6 – 177 g.m-2). *Stn. 48 had no available biovolume density data, however it was described to have a microbial community structure comparable to the Stn. 14, in which biovolume density was 0.2 $mm^3.cm^{-3}$, thus we classified Stn. 48 as low abundant. Map created using ESRI ArcGIS 10.8.1. Final layout of the figure was done in Inkscape 1.1.2 , www.inkscape.org.

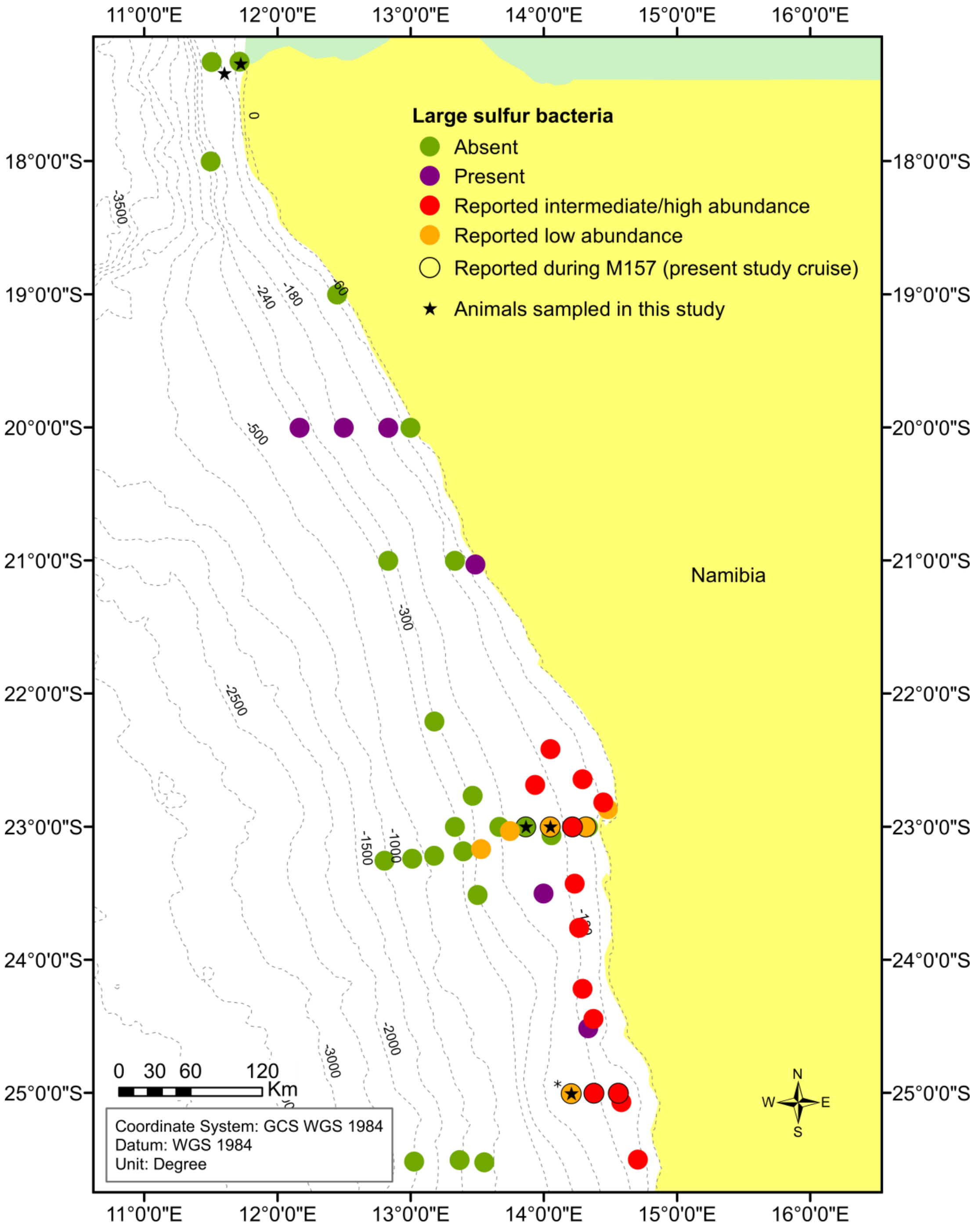

1 Neumann, A., Lahajnar, N. & Emeis, K. C. Benthic remineralisation rates in shelf and slope sediments of the northern Benguela upwelling margin. *Continental Shelf Research* **113**, 47-61 (2016).

2 Brüchert, V. *et al*. Regulation of bacterial sulfate reduction and hydrogen sulfide fluxes in the central Namibian coastal upwelling zone. *Geochimica et Cosmochimica Acta* **67**, 4505-4518 (2003).
